# Recurrent Lupus Enteritis While on Chronic Immunosuppressant Therapy

**DOI:** 10.7759/cureus.30149

**Published:** 2022-10-10

**Authors:** Nandhini Bindukumari Sureshkumar, Prathima Gopinath, Akanksha Joshi, Sreerag Alumparambil Surendran

**Affiliations:** 1 Internal Medicine, Marshfield Clinic Health System, Marshfield, USA; 2 Critical Care, Icahn School of Medicine at Mount Sinai, New York, USA; 3 Rheumatology, Marshfield Clinic Health System, Marshfield, USA; 4 Hospital Medicine, Marshfield Clinic Health System, Marshfield, USA

**Keywords:** sle enteritis, rare cause of acute abdominal pain, hydroxychloroquine, mycophenolate, bowel wall thickening, lupus enteritis, recurrent enteritis, immunosuppression, enteritis, sle

## Abstract

Lupus enteritis is a poorly studied cause of abdominal pain in patients with systemic lupus erythematosus (SLE). We present the case of a 28-year-old female with a history of SLE for nine years. She has been on chronic immunosuppressant therapy for the last nine years due to an episode of lupus enteritis in the past. Currently, the patient presented to urgent care with a three-day history of waxing and waning symptoms of abdominal pain, vomiting, and diarrhea. In addition, the patient had skin rashes. Laboratory work was significant for leukopenia, hypocomplementemia, hematuria, and proteinuria. CT of the abdomen showed bowel thickening involving the entire ileum, distal jejunum, and first portion of the duodenum. It was accompanied by moderate mesenteric edema and a small amount of ascites. Since the patient was on long-term immunosuppressive therapy with hydroxychloroquine and mycophenolate mofetil, infectious etiology was of high consideration; however, it was ruled out after further testing. Along with continuing her home dose of mycophenolate mofetil and hydroxychloroquine, the patient was started on IV methylprednisolone 1 mg/kg for three days. The patient dramatically responded to IV steroids. The patient was transitioned to oral prednisone 60 mg daily, and steroids were tapered off by 10 mg each week. A repeat CT scan in two months showed the resolution of the previously visualized small bowel wall thickening. This case highlights that chronic immunosuppression should not preclude differential or diagnosis of lupus enteritis in a patient with a history of SLE.

## Introduction

Systemic lupus erythematosus (SLE) is an autoimmune disease that affects multiple systems [1]. Literature has estimated wide variations in the incidence of gastrointestinal (GI) involvement with SLE. However, enteritis as a complication of SLE has high morbidity and mortality [2]. It usually presents with abdominal pain, vomiting, and fever [3]. However, when the patient is on chronic immunosuppressant therapy, physicians may overlook the possibility of lupus enteritis since the management of this type of enteritis is itself immunosuppression. There have been only a limited number of case reports on flare-ups of lupus enteritis in patients already receiving significant immunosuppressive therapy. We report a case where a patient had a relapse of lupus enteritis while being on the two commonly used immunosuppressive medications, mycophenolate mofetil and hydroxychloroquine.

## Case presentation

This is a 28-year-old Caucasian female with a history of SLE diagnosed nine years ago based upon her symptoms of polyarthralgia, myalgia, fatigue, fever, skin rash with biopsy consistent with lupus, low complement levels, positive antinuclear antibody (ANA), positive anti-double stranded DNA antibody (anti-ds-DNA), positive anti-Sjogren's syndrome A antibody (anti-SS-A), and positive anti-Sjogren's syndrome B (anti-SS-B) antibodies. The patient subsequently had an episode of lupus enteritis, and since then has been on long-term immunosuppressive therapy with hydroxychloroquine 200 mg two times a day and mycophenolate mofetil 1000 mg two times a day. She presented to the urgent care center with a three-day history of waxing and waning symptoms of abdominal pain, vomiting, and diarrhea. She was having six to seven episodes of watery stools a day without any hematochezia or melena. She denied having any sick contact, any change in eating habits, urinary frequency, or urgency. She did not complain of any associated skin rash, cough, respiratory symptoms, or joint pain. The patient reported compliance with all her medications.

**Figure 1:**
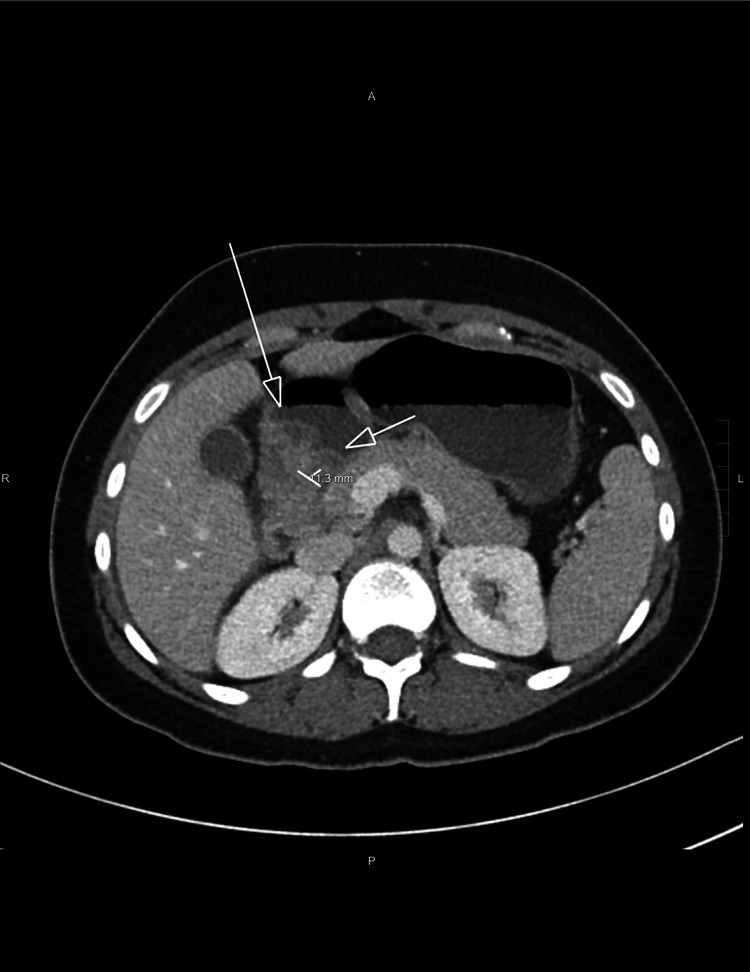
Abnormal wall thickening suggested at the first portion of the duodenum with rounded margins of the duodenal wall and thickening measuring up to 1.1 cm (portion between the arrows).

On presentation, her vitals were stable. The abdomen was soft to palpation but revealed an erythematous, blanching, macular rash around the umbilicus. Labs were relevant for leucopenia with a WBC of 2800/uL, normal lactic acid of 0.7 mg/dL, and a normal procalcitonin of 0.06 ng/mL. Tests for Clostridium difficile nucleic acid, stool white blood cell count, stool cultures, Salmonella, Shigella, and Campylobacter were all negative. The patient was tested negative for COVID-19 as well. The CT abdomen showed a marked abnormality involving the small intestine (Figure 1). There was a very long segment of moderate, contiguous small bowel wall thickening involving the entire ileum and distal jejunum. It was associated with moderate mesenteric edema and a small amount of ascites. Additionally, there was thickening of the first portion of the duodenum (measuring up to 1.1 cm) between the gastric antrum and ampullary region. Diffuse small bowel enteritis was suggested. Given the history of SLE, lupus enteritis was a favored diagnosis. The skin rashes and leukopenia added to this suspicion. However, since the patient was on chronic immunosuppressive therapy, the possibility of an underlying infectious etiology could not be ruled out. On further work-up, there was hypocomplementemia of C3 and C4 (C3 of 66 mg/dL and C4 of 10.6 mg/dL). The anti-dsDNA antibody was at the upper limit of normal. She also developed new-onset microscopic hematuria and proteinuria, which are concerning for renal involvement from lupus. Serum creatinine was normal at 0.6 mg/dL. Rheumatology was consulted. In addition to continuing with her home dose of mycophenolate mofetil and hydroxychloroquine, the patient was started on a high-dose steroid regimen with IV methylprednisone 1 mg/kg for three days. The patient was concomitantly placed on proton pump inhibitors twice daily for GI prophylaxis. An upper GI endoscopy (EGD) up to the third portion of the duodenum was done with biopsies to distinguish SLE enteritis versus infectious or other forms of inflammatory enteritis. EGD images were found to be normal up to the third portion of the duodenum. Biopsies did not reveal any evidence of ischemic injury but only showed mild nonspecific active duodenitis.

**Figure 2:**
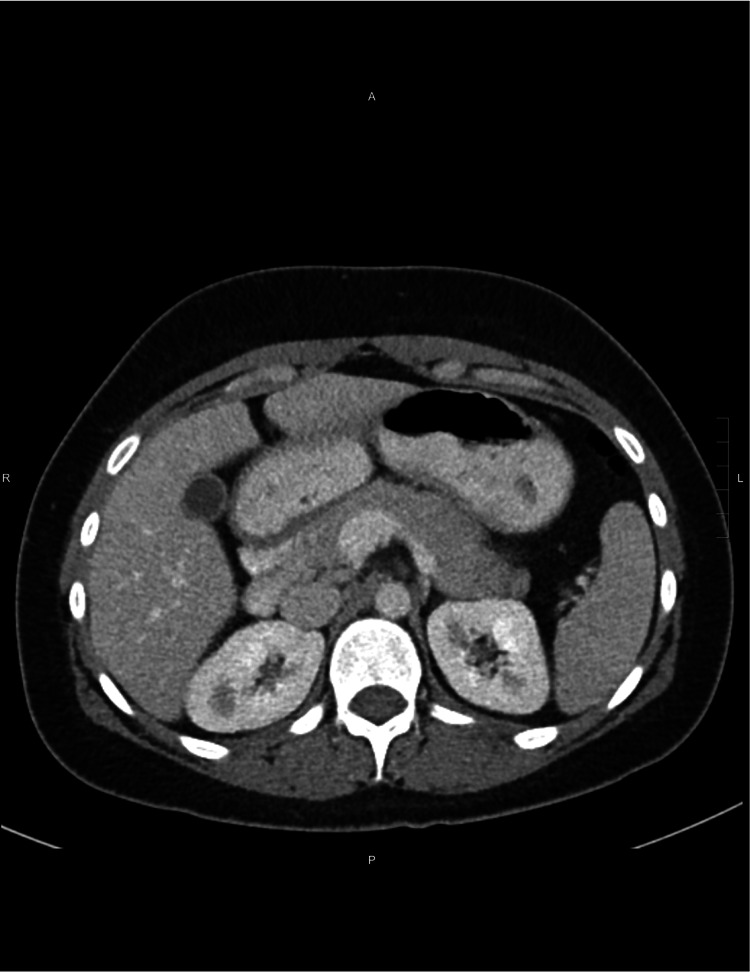
Repeat CT in two months shows resolution of previously identified small bowel wall thickening.

The patient started improving symptomatically, responding dramatically to IV steroids with no further episodes of nausea and vomiting. The erythematous macular rash on her abdomen, as well as the hematuria, started vanishing on the third day of her admission. The patient was switched to oral prednisone 60 mg daily on the fourth day. Prednisone was scheduled to taper by 10 mg each week. Since the administration of high-dose steroids, she has also been started on pneumocystis pneumonia prophylaxis with trimethoprim-sulfamethoxazole. She tolerated the diet well and was subsequently discharged from the hospital on the fifth day of hospitalization. She had a follow-up CT scan after two months as an outpatient, which showed resolution of the previously visualized small bowel wall thickening (Figure 2).

## Discussion

Gastrointestinal activity is not one of the 17 SLICC (Systemic Lupus International Collaborating Clinics) criteria for SLE [4]. SLE-related causes of acute abdominal pain include lupus enteritis, pancreatitis, pseudo-obstruction, acalculous cholecystitis, mesenteric thrombosis, hepatic thrombosis, colonic perforation from vasculitis, and medication-related pain (secondary to NSAID-induced gastritis or steroid-induced gastritis) [5]. Lupus enteritis is a rare cause of abdominal pain in SLE patients. It might occur even in patients whose disease activity is under control [6]. If left untreated, it can lead to significant complications such as intestinal necrosis and perforation [7]. Hence, it is necessary to have a high index of suspicion for timely diagnosis based on the clinical picture and imaging [5].

Lupus enteritis most commonly affects the jejunum and ileum [6]. However, it sometimes has rectal involvement as well [6]. One proposed mechanism of lupus enteritis is the activation of complements, leading to diffuse microvascular injury. This results in increased vascular permeability, eventually leading to interstitial edema [6].

A CT scan is the gold standard for the diagnosis of lupus enteritis [8]. Three classic patterns suggestive of lupus enteritis are bowel wall thickening greater than 3 mm ("target sign"), engorgement of the mesenteric vessels ("coombs sign"), and increased attenuation of mesenteric fat. Arteriography may reveal arterial narrowing and distended loops of the bowel [8]. However, the lack of specificity of patterns found in CT scans is a limitation [2], as these signs can be seen in pancreatitis, mechanical bowel obstruction, peritonitis, and inflammatory bowel disease [2]. The differential diagnosis of edematous and thickened small intestinal walls includes Henoch-Schönlein purpura, intestinal anisakiasis, and eosinophilic gastroenteritis [9]. The yield from the biopsy is limited to a diagnosis of lupus enteritis [8].

Lupus enteritis is generally reversible and steroid-responsive in the acute setting [2]. Hydroxychloroquine, mycophenolate mofetil, azathioprine, and low-dose steroids are usually used for long-term maintenance [2]. However, in our case, the patient developed a recurrence of lupus enteritis while on mycophenolate mofetil and hydroxychloroquine. A regimen used for lupus nephritis, using the Euro lupus nephritis trial, was effective in one case report [10]. The regimen was low-dose intravenous cyclophosphamide (a cumulative dose of 3 g) followed by azathioprine [10].

There are no controlled studies on what to use in recurrence. In a limited number of cases, mycophenolate mofetil, cyclophosphamide, and rituximab have been successfully used to prevent further recurrences [2].

## Conclusions

We report this case to make physicians aware that lupus enteritis can recur even when the patient is on chronic immunosuppressant medications like hydroxychloroquine and mycophenolate mofetil. Differentials are wide in a patient presenting with abdominal pain who is immunocompromised, including the possibility of infectious etiology. Once infectious etiologies are ruled out, it is pertinent to consider lupus enteritis in a patient with a history of systemic lupus erythematosus. With a lack of evidence or controlled studies on which regimen works best to prevent recurrences, this remains a potential knowledge gap for the care of patients with lupus enteritis.
